# Fracture Risk Assessment in Metabolic Syndrome in Terms of Secondary Osteoporosis Potential. A Narrative Review

**DOI:** 10.1007/s00223-025-01341-5

**Published:** 2025-02-20

**Authors:** Ferah Armutcu, Eugene McCloskey

**Affiliations:** 1 Sanctuary International Visitor Support Scheme, University of Sheffield, Sheffield, UK; 2 Division of Clinical Medicine, School of Medicine and Population Health, University of Sheffield, Western Bank, Sheffield, S10 2TN UK; 3 Versus Arthritis Centre for Integrated Research in Musculoskeletal Ageing (CIMA), Mellanby Centre for Musculoskeletal Research, University of Sheffield, Sheffield, UK

**Keywords:** Metabolic syndrome, Obesity, Hyperglycemia, Insulin resistance, Secondary osteoporosis, Fracture risk assessment

## Abstract

Osteoporosis is a major global public health problem with the associated bone fractures contributing significantly to both morbidity and mortality. In many countries, osteoporotic fractures will affect one in three women and one in five men over the age of 50. Similarly, diabetes, obesity, and metabolic syndrome (MetS) are among the leading public health problems due to their worldwide prevalence and burden on health budgets. Although seemingly disparate, metabolic disorders are known to affect bone health, and the interaction between fat and bone tissue is increasingly well understood. For example, it is now well established that diabetes mellitus (both type 1 and 2) is associated with fracture risk. In this narrative review, we focus on the potential link between MetS and bone health as expressed by bone mineral density and fracture risk. This narrative review demonstrates the association of MetS and its components with increased fracture risk, and also highlights the need for fracture risk assessment in patients with obesity and MetS.

## Introduction

Metabolic syndrome (MetS) is characterized by the co-occurrence of several common abnormalities, including high blood pressure, atherogenic dyslipidemia (high triglycerides levels and reduced HDL levels and), high blood glucose, insulin resistance (IR), and central obesity [1]. The main utility of diagnosing MetS is in identifying individuals at high risk of developing cardiovascular disease and type 2 diabetes mellitus (T2DM). Much research has exposed an association between diabetes mellitus and fracture risk, with the conclusion that both type 1 and T2DM increase the risk of fracture [2]. At first, this is perhaps surprising in the context of T2DM where body mass index (BMI) and bone mineral density (BMD) are also characteristically higher in affected versus unaffected individuals. However, fracture risk in T2DM is greater than that predicted from these and other risk factors used in tools such as the fracture risk assessment tool FRAX [3] (https://frax.shef.ac.uk/FRAX/) leading to some suggestions about how the excess risk arising from T2DM can be incorporated in FRAX. These include reducing the T-score by 0.5, adding 10 years to the patient’s age, including ‘rheumatoid arthritis’ as a comorbidity representing T2D, or adding a trabecular bone score adjustment [4]. Given that MetS is a precursor of T2DM, the question arises if fracture risk is also increased in MetS, the global prevalence of which is significantly greater than that of T2DM. For example, in a recent prevalence pooling meta-analysis using random-effects models, the global prevalence of MetS was greater than 40% for ethnic-specific central obesity, hypertension, and low HDL cholesterol [5]. Increased serum triglycerides or increased fasting glucose was reported in 20–30% of individuals. In contrast, the prevalence of diabetes mellitus worldwide in the adult population is assumed to be 6059 cases per 100,000 [6]. The main aim of this narrative review is to provide comprehensive and up-to-date information on the risk of osteoporotic fractures in patients with MetS and the indication for potential risk assessment. For this purpose, scientific studies between 1996 and 2024 were searched using MEDLINE, PubMed, and Google Scholar. The relevant web searches mostly used the terms ‘Metabolic syndrome’; ‘bone fracture risk’; and additional keywords such as ‘abdominal obesity,’ and ‘secondary osteoporosis’ were combined with these two keywords.

### Metabolic Syndrome and Bone Health

**Fig. 1:**
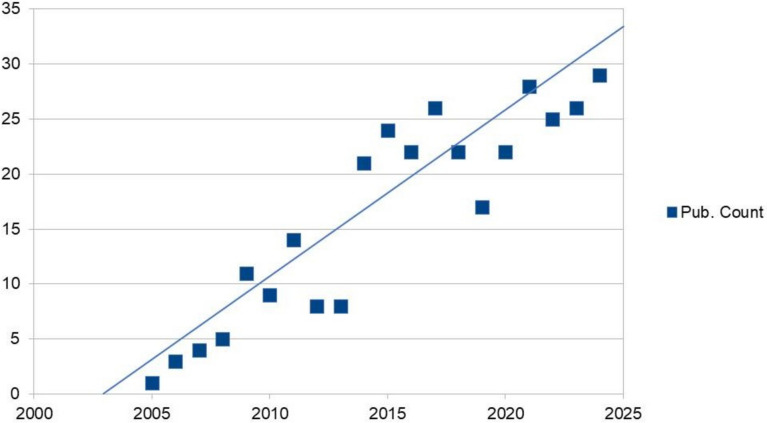
The graph represents the linear increase in the number of publications on the relationship between ‘abdominal obesity and bone health’ in PubMed between 2005 and 2024. Search query keywords ‘abdominal obesity and bone health’ (Data was extracted on 4th Oct 2024)

MetS can affect bone health in different ways, and the relationship between the two is complex. In addition to factors that may increase the risk of low BMD in MetS, such as hormonal and biochemical changes, inflammatory and oxidative environment, and mechanical loading, gender difference, and health behaviours such as smoking and alcohol consumption are also important [7–9]. MetS and its components, which are associated with important public health problems with high prevalence, especially obesity and diabetes, may contribute to the etiopathogenesis of many diseases from cardiovascular diseases to cancer [10, 11]. The results of previous meta-analyses examining the association between MetS and bone fracture risk suggest that the latter is not directly affected by MetS, or if an effect was observed then MetS was associated with a lower risk of fracture (without adjustment for BMD) [12–14]. The importance and uncertainty about the relationship between MetS and osteoporosis, similar to the relationship between obesity and osteoporosis, has resulted in a remarkable linear increase in studies on ‘obesity and bone health’ in the last two decades (Fig. 1). Several studies suggest an increased risk of osteoporosis and/or fractures in MetS, with a suggestion of possible gender-based differences. For example, in a study of European Caucasian women, a significant association was shown between MetS and low BMD [15], but the same authors reported no such association in a study of Caucasian men [16]. In another population-based study, women with MetS were reported to have a higher risk of fracture compared with men with MetS [17]. A recent meta-analysis suggested that bone mass is normal in men with MetS [18], while a further cohort study in 117,000 individuals concluded that hyperglycemia significantly increased fracture risk but only in women [19]. Hypertriglyceridemia has been associated with an increased risk of hip fracture in men [19, 20], but there are also studies suggesting that this association is not significant or showing conflicting data depending on gender [21–23]. According to Babagoli and colleagues [24], who reported that MetS had a protective effect on bone fracture rates in men with no clear effect on fractures in women, the lack of an association between MetS and increased fracture risk in the general population may be explained by the fact that MetS is not a single pathological entity. The relationship between the various components of MetS and bone health is reviewed .

### Central or Abdominal Obesity

Obesity, a feature of MetS, is associated with chronic inflammation, and abdominal obesity is also considered a marker of dysfunctional adipose tissue, which contributes to the pro-inflammatory state associated with MetS [25]. Recent data supporting a positive association between BMI and BMD suggest that obese individuals generally have lower bone turnover and greater bone strength [26]. Meta-analyses of prospective cohort studies have shown that obesity is associated with a significant reduction in the risk of hip fracture [27]. However, the relationship between obesity and fracture risk is more complex than it first appears [28]. For example, in a meta-analysis of international cohorts [29], a BMI of 35 kg/m2 was associated with a 13% decrease in osteoporotic fracture risk compared to women with a BMI of 25 kg/m2 [(hazard ratio (HR), 0.87; confidence interval (CI), 0.85–0.90]. When adjusted for BMD, however, the same comparison showed that the HR for osteoporotic fracture was actually increased at the higher BMI (HR, 1.16; CI, 1.09–1.23) [29]. Internationally applicable fracture risk assessment tools, such as FRAX, use BMI in their risk calculations as this adjusts, to a reasonable extent, for international variations in height and weight. However, BMI does not distinguish between excess fat, muscle, or bone mass, nor does it provide any indication of the distribution of fat within individuals. The latter is of particular importance as it has long been recognized that BMI fails to fully capture cardio metabolic risk which relates more to abdominal adiposity, a key risk factor in MetS. Waist circumference (WC), as the clinical diagnostic standard of central obesity, is an important indicator for MetS and is strongly associated with all-cause and cardiovascular mortality, with or without adjustment for BMI [30].

Some meta-analyses have shown that high abdominal obesity may be detrimental to bone health when adjusted for BMI [31, 32]. For example, in the Norwegian Cohort study, Søgaard and colleagues followed a population of 19,918 women and 23,061 men aged 60–79 years for an average of 8.1 years [33]. As reported in other analyses, hip fracture risk decreased with increasing BMI, but higher WC and higher waist-to-hip ratio were associated with increased hip fracture risk after adjustment for BMI and other possible confounders. The increased risk of hip fracture in the highest tertile of WC, compared to the lowest, was similar in women and men (86% increase, 95% CI: 51–129% vs 100% increase, 95% CI 53–161%, respectively). Furthermore, lower BMI combined with abdominal obesity increased the risk of hip fracture considerably, particularly in men. In a meta-analysis of up to 9 studies, with a total sample size of almost 300,000 individuals (129,964 men and 165,703 women), Sadeghi reported that abdominal obesity (defined by various waist–hip ratios) was positively associated with the risk of hip fracture (CI, 1.24; 1.05–1.46, P = 0.01), with a similar but not statistically significant effect seen when using WC (RR: 1.36; 95% CI: 0.97–1.89, P = 0.07) [34]. A 0.1-unit increase in waist–hip ratio was associated with a 16% increase in the risk of hip fracture (relative risk RR, 1.16; CI, 1.04–1.29, P = 0.007), whereas a 10-cm increase in WC was not significantly associated with a higher risk (1.13, 95% CI: 0.94–1.36, P = 0.19) [34]. In a separate but overlapping meta-analysis population involving up to 200,000 individuals, Li and colleagues reported RRs between the highest and lowest categories of 1.58 (CI, 1.20–2.08) and 1.32 (CI, 1.15–1.52) for WC and waist–hip ratio, respectively [35]. Hip fracture risk appeared to increase by 3% for each 0.

1 unit increment of waist–hip ratio (RR, 1.03; CI, 1.01–1.04), whereas a higher hip circumference was associated with a trend to reduce hip fracture risk (RR, 0.87; CI, 0.74–1.02) [35].

More recently, Zhu and colleagues published an analysis of prospective data from the UK Biobank study comprising 205,029 men and 241,750 women with a mean age of 57 years (range 38–79 years), of whom 2.22% sustained incident fractures (excluding those of the skull, face, hands, and feet, pathological fractures, atypical femoral fractures, and periprosthetic fractures) over just under 8 years of follow-up [36]. In a linear model, higher BMI played a protective role for fracture, when adjusted for age, sex, smoking and drinking status, regular physical activity, the use of glucocorticoids, socioeconomic status, and processed meat intake (HR, 0.99; p = 0.0011). However, in a restricted cubic spline analysis, a U-shape association was observed between BMI and fracture risk with the lowest risk of fracture being observed in those with BMI in the overweight category (25.0–29.9 kg/m2). In contrast to those who were overweight, when adjusting for the aforementioned covariates and falls, the risk of fracture was higher in underweight participants (HR, 1.57; CI, 1.19–2.06). When additionally adjusting for BMD in both sexes, this effect was more pronounced in men than in women. However, fracture risk was significantly increased in obese subjects when adjusted for BMD, while waist circumference adjusted for BMI also had a linear association with fracture risk in both men and women (HR, 1.02; CI, 1.01–1.02) [36]. Likewise, another study suggested that larger WC and/or higher BMI were significantly associated with increased fracture risk at specific skeletal sites [37]. In the GLOW study, an increased incidence of fracture at the ankle and upper leg was noted in obese compared to non-obese women, while the risk of wrist fracture was significantly lower. Furthermore in the CARTaGENE cohort, significant relationships were found between WC and distal lower limb fractures in individuals with BMI that were normal or overweight, but not in those in the obesity category [38]. In the overweight category, an increased risk of distal upper limb fractures with increasing WC was also noted.

Interestingly, several studies show that a higher WC, adjusted for BMI, is also associated with a lower BMD than expected [32, 36]. That the discordance between BMI and WC is likely due to increased abdominal fat tissue may underpin this observation. For example, in a recent study of almost 11,000 participants aged 20–59 years from the NHANES cohort [39], a difference in the direction of the relationship was observed between BMD and either lean mass index (LMI, lean mass divided by height squared) or fat mass index (FMI, fat mass divided by height squared). Thus, in multivariate analyses, every 1 kg/m2 increase in LMI was associated with a 0.19 higher T-score, while every additional 1 kg/m2 increase in the FMI was associated with a 0.10 lower T-score (P < 0.001 for both). Effects of LMI were similar in men and women, whereas the increase in FMI was associated with a lower BMD in men than in women (0.13 vs 0.08 T-score, respectively, p < 0.001) [39]. In a subsequent analysis, examining the relationship between BMD and compartments of adipose tissue, the same study showed a strong negative effect of visceral adipose tissue on BMD; in an adjusted model, each higher quartile of VAT was associated with an average 0.22 lower T-score (CI, − 0.26 to − 0.17) [32]. A study from Korea also demonstrated a negative correlation between lumbar spine BMD and waist–hip ratio [40].

### Insulin Resistance

The relationship between IR and bone health has recently been reviewed [41] and is, therefore, only addressed briefly here. Simply defined as an impaired biological response to insulin stimulation in target tissues, IR is primarily related to liver, muscle, and adipose tissue [42]. Hyperinsulinemia, associated with pancreatic islet hyperplasia, frequently precedes obesity and diabetes in MetS and is, thus, considered an early indicator of metabolic dysfunction [43]. Hyperinsulinaemia is believed to promote bone formation through pro-osteoblastic mechanisms and has traditionally been associated with increased bone mass. While some studies reported that this positive association was independent of BMI [44, 45], others noted that the association was lost after BMI adjustment [46–49]. For example, in the MIDUS II study, an inverse relationship was shown between the homeostatic model assessment of IR (HOMA-IR) and calculated indices of bone strength [47]. In another study, a positive correlation was observed between HOMA-IR and total volumetric BMD, trabecular vBMD, and trabecular thickness but a negative correlation was found with bone size [50]. Recently a longitudinal study data has suggested adolescent IR may be detrimental to bone development through puberty, independent of body composition and the level of physical activity [51]. Interestingly, following adjustment for higher BMD and BMI in non-diabetic elderly, higher IR tended to be associated with an increased risk of fracture, although not statistically significant [48]. Thus, while current evidence suggests that insulin has favourable anabolic effects on bone, it also suggests that IR negatively affects bone structure and quality. One mechanism by which IR might influence the latter is through effects on bone turnover which is reported to be lower in patients with IR [41, 52, 53].

### Low HDL and Elevated Triglycerides

A key component of MetS, dyslipidemia comprises the triad of elevated levels of small dense low-density lipoproteins (sdLDL) and triglycerides, coupled with lowered levels of cardio protective high-density lipoproteins (HDL). Its pathogenesis appears to be driven by IR, dysfunction of white adipose tissue and chronic energy imbalance [54]. While there is a well-established relationship between dyslipidemia and a higher risk of cardiovascular events, the relationship between dyslipidemia and its components with BMD and fracture risk is less certain. For example, with regard to BMD, an early study in women age 50–59 years reported a positive association between elevated triglycerides and BMD, but a negative association of BMD with HDL-C [55]. In contrast, a Korean study in older adults (men over 50 years and postmenopausal women) using KNHANES data (2008–2011) found that serum triglycerides had a negative association with whole-body BMD [56]. In a recent study investigating the association of multiple lipid metabolism indicators and bone health in 380 Chinese subjects, lipid metabolism indices were positively or negatively correlated with BMD to varying degrees [57]. In women, elevated levels of triglycerides, total cholesterol (TC) and low-density cholesterol (LDL-C) were associated with a lower BMD. In contrast, a largely opposite effect was seen in men; for example, higher LDL-C correlated with higher BMD. Inconsistent results examining the relationship of HDL-C with BMD have been reported in other studies [58–61].

With regard to fracture risk, the picture also remains somewhat unclear. In an analysis of the Tromso study, no association was observed between TG levels and fracture risk in men or women, but higher HDL-C was linked to a higher fracture risk in women, and in men with a higher BMI [62]. Another cohort study also reported that elevated levels of HDL-C were linked to incident fractures in both male and females, irrespective of traditional risk factors [63]. Finally, a prospective observational study of men and women included in the Cardiovascular Health Study also reported no association with TG levels, but noted that HDL-c and LDL-c levels had statistically significant non-linear U-shaped relationships with hip fracture risk (HDL-c, p = 0.009; LDL-c, p = 0.02). In fully adjusted conjoint models, higher VLDL particle concentration and size, and higher HDL-C particle size were associated with higher hip fracture risk [64]. In contrast, in the SWAN study of midlife women, high fasting triglyceride levels (≥ 300 mg/dl) had about a 2—to 2.5-fold increased risk of non-traumatic fractures, after controlling for potential confounders such as BMD and BMI [65]. However, no associations were observed between total cholesterol, LDL-C, or HDL-C levels and fractures. In a study of Korean men, none of the individual measures of dyslipidemia were significantly associated with fracture risk; the latter tended to be lower in those with individual components present, but this was largely explained by a higher BMI in those with MetS [66]. A similar protective association between TG levels and fracture risk was reported in men from the MINOS study, despite the men with MetS having lower BMD attributed to abdominal obesity [67].

### Hypertension

**Fig. 2:**
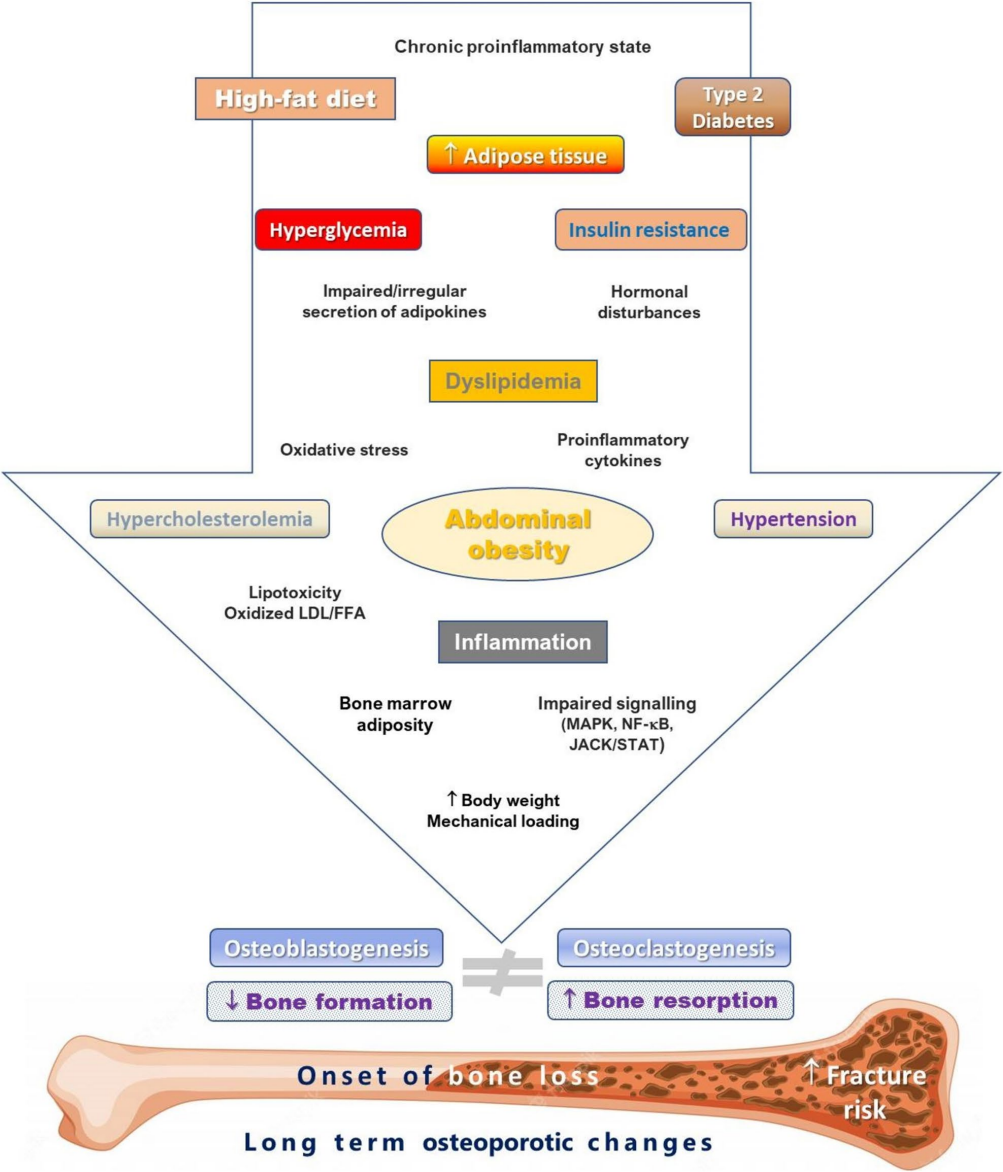
Primary (framed) and secondary factors in the effects of metabolic syndrome components on bone health

For more than 30 years, the primary mediators of hypertension in MetS and obesity are thought to be overstimulation of the sympathetic nervous system, IR, and increased renal sodium reabsorption due to hyperinsulinemia [68]. Another compelling link between obesity and vascular diseases such as hypertension and T2DM is increased adiposity [69]. Analysis of body composition using dual X-ray densitometry (DXA) showed that the relationship between fat mass and lean body mass was altered in hypertensive adolescents [70]. It has been suggested that changes in the adipokine profile due to nutrient excess and increased pro-inflammatory cells lead to an increase in perivascular adipose tissue inflammation and impaired vascular function [71]. Naturally, hypertension is also independently associated with osteoporosis [72]. Both hypertension and osteoporosis have a common underlying dietary aetiology in terms of dietary salt intake, so sodium is the main factor linking blood pressure and osteoporosis [73]. There is a strong link between salt consumption and blood pressure, and it is also thought that patients with high blood pressure excrete more calcium in the urine and, therefore, have a higher risk of developing osteoporosis [74]. According to Hong et al., low sodium intake was associated with osteoporosis [75]. However, an experimental study showed that a long-term excessive salt consumption accelerated bone loss in rats [76]. Likewise, higher sodium intake has been found to be associated with a higher prevalence of osteoporosis in postmenopausal women [77]. Hypertension also increases urinary calcium excretion, which is an important factor affecting calcium metabolism and, thus, bone homeostasis [8, 78]. For example, hypertensive osteoporotic women had a significantly higher BMI-adjusted calciuria and calcium/creatinine ratio compared with non-hypertensive osteoporotic women [79]. On the other hand, also hyperactivity of the hypothalamic–pituitary–adrenal axis and hypercortisolism may lead to decreased levels of bone formation; for example, a cross-sectional study has shown that hypertension is negatively correlated with bone formation in patients with newly diagnosed osteoporosis [80]. Ultimately, various changes in body physiology noticed in hypertensive individuals, such as increased sympathetic tone, altered renin–angiotensin–aldosterone system, oxidative stress, and increased levels of certain cytokines, are known to drive bone remodelling towards increased bone resorption [81]. Figure 2 provides an overview of the main and secondary drivers of the effects of MetS components on bone health.

### Medications Targeting both Metabolic Syndrome and Bone Health

In patients with MetS and abdominal obesity, weight loss through dietary modification and physical exercise is an essential element of treatment against the risk of osteoporosis. However, it should be recognized that excessive weight loss may damage bone tissue, as mechanical loading is important in maintaining bone health [82]. The use of pharmacological agents in addition to exercise and lifestyle changes in the treatment of MetS is often targeted at MetS components. Improvements in patients’ glycemic status, lipid profile, and blood pressure will contribute to a reduction in inflammation and oxidative status and, thus, bone health [9]. For example, palmatine, a naturally occurring isoquinoline alkaloid, may protect against both complications of MetS such as cardiovascular disease and osteoporosis, with these protective effects largely attributed to its antioxidant and anti-inflammatory properties [83]. Against hyperlipidemia, a component of MetS, 3-hydroxy-3-methylglutarylo-CoA (HMG-CoA) reductase inhibitors (statins) are the most commonly prescribed lipid-lowering agents [9, 84]. Preclinical and clinical study results suggest that these agents also have potential beneficial effects on bone metabolism [85]. Statins inhibit osteoclastic activity by reducing the production of downstream products such as farnesyl pyrophosphate and geranylgeranyl pyrophosphate in the HMG-CoA blockade mevalonate pathway [86]. The same pathway is shared by nitrogen-containing bisphosphonates, which are potent inhibitors of bone resorption, so that bisphosphonates indirectly also prevent the prenylation of Rho family GTPases, which are essential for the function and survival of bone-resorbing osteoclasts [87]. Another mechanism of statins is that they act as osteoclastogenesis inhibitors by suppressing ROS-mediated signalling pathways. In other words, they inhibit RANKL, which is required for osteoclast differentiation, by inhibiting ROS production [88]. Although anti-diabetic agents are generally preferred against hyperglycemia, it should be kept in mind that they may have different effects on bone remodelling [89].

Almost 80% of patients with MetS have hypertension; calcium channel blockers have a neutral effect on MetS, while inhibitors of the renin-angiotensin system are thought to provide the most benefit. Although the use of thiazide diuretics and beta-blockers is not recommended in the population with MetS, new evidence suggests that they may be used under certain conditions [90]. A new observational study has revealed a strong association between hypertension and a higher prevalence of osteoporosis [91]. This highlights the need to develop timely and effective preventive strategies and treatment modalities to reduce the prevalence and burden of disease in hypertensive individuals at risk of osteoporosis. For example, in elderly hypertensive patients, the incidence of osteoporotic fractures has been reported to decrease as the number of daily antihypertensive medications increases [92].

The treatment of diabetes and osteoporosis often involves a combination of lifestyle changes, physical activity, and medications. Indeed, medication used to treat the combination of diabetes (or prediabetic conditions such as MetS and obesity) and osteoporosis includes bisphosphonates, anabolic agents, metformin, GLP-1 analogues, anti-sclerostin antibodies and hormone replacement therapy. It is notable the impact of GLP-1 agonists on bone metabolism and the risk of fractures [93]. Treatment with a GLP-1 antagonist reduces bone resorption by affecting the balance between osteoclasts and osteoblasts. For example, the anabolic effect of liraglutide on GLP-1 receptors in pre-osteoblasts and osteocytes suggests that it may reduce fracture risk by preventing the loss of bone mass associated with weight loss [94]. Mature osteocytes with GLP-1 receptors also produce sclerostin, which inhibits Wnt/β-catenin signalling by binding with LDL-receptor-related protein 5 and preventing Wnt binding [95]. A meta-analysis study found that liraglutide and lixisenatide statistically significantly reduced the risk of fractures compared with placebo and other anti-diabetic drugs, and that their beneficial effects were dependent on the duration of treatment [96], while another recent study reported that liraglutide is an effective weight loss strategy that also preserves bone health during weight loss in women with obesity [97].

### Discussion

Both MetS and osteoporosis are two public health problems worldwide, particularly affecting the ageing population aged 50 years and over. While there is good evidence of the interplay between MetS and bone, including fracture risk, the overall effect is complex, probably reflecting the fact that MetS is not a single pathological body [29]. Recent studies have reported that each component of MetS is associated with poor skeletal health, including hyperglycemia [98], dyslipidemia [84], and hypertension [78]. Indeed MetS combines several components that have different and sometimes opposite effects on bone health and fracture risk. Inevitably, these results also raised the suspicion that there may be sexual dimorphism in the clinical expression of MetS and osteoporosis recorded in the same geographical area and over the same period. MetS is known to promote systemic inflammation and induce hormonal changes that negatively affect bone health [99, 100]. However, the assessment of the potential impact of MetS and obesity on fracture risk is a complex issue and may also vary depending on individual factors [101]. Some study results report that MetS is associated with an increased risk of low BMD [20, 102], whereas others suggested that MetS is associated with a lower risk of osteoporosis [103, 104] or no correlation [105, 106]. In 2016, Qin et al. [107] reported that the presence of MetS was significantly associated with a recent history of osteoporotic fracture in a large sample of middle-aged and elderly Chinese women. They also suggested that central obesity seems to have a strong association with the prevalence of osteoporotic fractures in women. According to a nested case–control study in South Korea, although MetS showed a low prevalence of osteoporosis, it was associated with a high risk of osteoporosis in both obese men and postmenopausal obese women [104]. Taiwanian Biobank study results showed that MetS could increase the risk of severe low bone density, and this risk could be minimized through higher BMI, non-smoking, no alcohol consumption, and regular exercise. Conversely, smoking, alcohol consumption, and lack of regular exercise could exacerbate the risk of severe low bone density. These findings highlight the importance of a multifactorial approach in managing bone healthcare [108]. Likewise, there are also studies showing that the relationship between MetS and bone health varies according to gender and population [18, 67]. In addition, hyperglycemia and oxidative stress, which are more common in people with MetS, as people age, can cause advanced glycation end-products (AGEs) to build up in bone. AGEs can alter the organic matrix, water, and mineral content, which can lead to bone fragility and a higher risk of fractures [109].

It has been projected that more than 319 million people globally will be considered to be at high risk of fragility fracture by the year 2040. Furthermore, individuals at high risk of osteoporotic fractures represent a significant disease burden for society worldwide, and this burden is projected to increase significantly in the future [110]. Therefore, risk assessment may provide a platform to evaluate prevention and intervention methods in patients at risk for osteoporotic fractures, such as diabetes, obesity, and MetS. Various fracture risk assessment tools have been developed to provide the basis for the integrated use of validated clinical risk factors to aid fracture risk prediction. FRAX®, a simple-to-use fracture risk tool, calculates the 10-year probability of a major osteoporotic fracture and hip fracture to guide clinical decision-making [3, 111]. This risk assessment tool integrates well-validated risk factors for fragility fracture with or without the use of BMD, calibrated according to the country-specific epidemiology of hip fracture and mortality [112, 113]. Criticized over the years for its limited number of risk factors and level of detail, FRAX, indeed was designed to be a simple, accessible, and easy-to-use tool in primary care [114]. Since most questions in the tool only have yes or no answers, it cannot be said that the number and dose-related risk factors are fully captured such as the number of prior fractures, the consumption of alcohol, and the dose of glucocorticoids. In addition, the lack of provision for lumbar spine BMD and the absence of measurements of the material or structural properties of bone are other concerns. Place of origin can also affect FRAX probabilities, as shown in a study in Sweden [115], where the hip fracture incidence for Swedish-born people was approximately double when compared to the one of people born outside the country. Additionally, FRAX with and without BMD was reported to be unaffected by body composition [116] and current or previous osteoporosis treatment [117]. Consequently, the main limitation of the previous FRAX tool was that the inputs were binary. Recently, the new FRAXplus tool has been developed to address many of these concerns and it is more preferred nowadays [118].

Unlike the traditional FRAX risk assessment application, FRAXplus® provides additional information for fracture risk probabilities, including recentness of prior fracture, exposure to high-dose oral glucocorticoids, duration of T2DM, trabecular bone score, recent falls history, and concurrent data on lumbar spine BMD and hip axis length. DXA-derived measures such as WC and waist-to-hip ratio or, when available, FMI and visceral adipose tissue can be used, although it remains unclear which measure to use in clinical settings. The latter usually require whole-body composition scans which are not frequently conducted in routine clinical practice. DXA scanners do, however, capture measures of abdominal tissue thickness as part of lumbar spine BMD measurement. In a recent analysis, and using Lunar DXA scanners, Leslie and colleagues [119] examined the impact of discordance between estimated abdominal thickness, derived from BMI, and actual measured abdominal thickness in over 73,000 individuals, with a mean age of 64.2 years. The authors suggested that increased abdominal thickness beyond that predicted by BMI and sex is a FRAX-independent risk factor for fracture, and this risk may be particularly important in individuals younger than 65 years. Although some guidelines base the initiation of fracture prevention therapy on the probability of a fracture estimated by FRAX [120], the utility of FRAX in women aged < 65 years is limited because it does not take into account menopausal status or the use of replacement therapy [121]. Indeed, the results of two studies, both using the FRAX tool, showed that women with MetS had a higher rate of fracture risk, and bone fracture risk may be different in men and women [17], similarly with an increased risk of bone fractures in middle-aged Korean women with MetS [122]. In practice, each patient should always be evaluated within their unique clinical context. Therefore, the use of FRAX may contribute to preventive medicine in terms of quantitative, personalized risk estimates to guide treatment decisions [123]. BMI is an important confounder interfering with FRAX risk assessment in patients with MetS in regression analysis [17]. This situation, which also poses a challenge for personalized fracture risk assessment in patients with MetS, can be overcome by using waist circumference, an indicator of abdominal obesity, in the FRAXPlus tool. As in T2DM, the association between MetS and fracture risk is unlikely to be driven by changes in bone density. Indeed, T2DM is associated with higher BMI and higher BMD, but a paradoxically increased risk for major osteoporotic fractures [124]. To adjust for the increased risk of fracture in T2DM, an International Osteoporosis Foundation working group recommends checking ‘yes’ on the rheumatoid arthritis input in patients with T2DM [124, 125].

Current evidence suggests that there is a significant economic burden associated with osteoporosis and osteoporotic fractures. Early diagnosis and treatment of patients at high risk of fracture is also extremely important for secondary prevention and reduced mortality as well as public health budget [126]. Regarding in terms of preventive medicine practices, fracture risk assessment in people with any metabolic disease such as obesity and MetS that contributes to fracture risk is very important in terms of reducing public health workload and health budget burden. This evidence indicates that a measure of abdominal obesity could improve or modify the prediction of fracture risk by tools such as FRAX in patients with MetS. Identifying patients at high risk for secondary osteoporosis, such as T2DM and obesity, as well as patients with MetS who are potentially at high risk, and adopting early and effective fracture prevention strategies are critical to reduce the burden of osteoporosis on health services. On the other hand, each of the methods proposed to address limitations in FRAX’s ability to assess fracture risk in individuals with T2DM has been found to improve performance [4]. In addition, a cohort study of middle-aged women, prediabetes before the menopausal transition were associated with a greater risk of fracture during the menopausal transition and after menopause, independent of BMD [127]. As in patients with diabetes, none of the tools available to assess fracture risk in patients with MetS can assess fracture risk comprehensively and multidimensional. Therefore, multiple methods can be combined in clinical practice to early identify patients at risk of osteoporotic fractures [128]. The complex relationship between MetS and fracture risk is also influenced by health behaviour factors. A recent large population-based cohort study has shown that MetS is associated with a risk of severe BMD and that certain health behaviours, such as smoking, alcohol consumption, and lack of regular exercise, are linked to the risk of low BMD [111]. Furthermore, studies supporting that osteoporotic fracture risk increases with increasing waist circumference [35, 36] suggest that abdominal obesity is not only associated with low BMD, but also systemic inflammation is associated with high fracture risk [129]. In addition to its high prevalence, the association of MetS and its components with bone health requires that this disease is addressed within the framework of an action plan. Therefore, FRAX risk assessment may be recommended as a priority target in terms of preventive medicine in obese women aged 45 years and over with a family history of diabetes and in men aged 55 years and over with abdominal obesity.

## Conclusion

Current literature and this review inspire that the potential risk of osteoporosis and fractures can be estimated using the FRAX algorithm in patients with MetS. As seen in Table 1, the factors associated with the development of osteoporosis and an increased risk of bone fracture overlap with the parameters used to determine the FRAX score. The potential benefits of FRAX, which forms the basis of population screening approaches for high fracture risk, particularly in primary care, cannot be overlooked. Therefore, considering the recommendations of the World Health Organisation to reduce the risk of osteoporotic fractures, it may be considered to routinely perform risk analyses using FRAX or similar tools in individuals living with obesity and MetS, principally in primary care [130, 131]. However, due to the limited number of existing studies on the relationship between MetS and osteoporosis, there is a need for more comprehensive and advanced studies with homogeneous study groups representing the entire population.

**Table 1:** Many factors related to the development of osteoporosis are directly or indirectly associated with FRAX clinical risk assessment factors

Factors affecting the development of osteoporosis	Clinical risk factors for FRAX assessment
- Genetic variation	- Height (cm)
- Peak bone mass	- Weight (kg)
- Vitamin D status—sunlight exposure	- Age
- Calcium intake	- Gender (Male/Female)
- Exercise	- Parent fractured hip
- Alcohol use	- Previous fracture
- Cigarette smoking	- Femoral neck BMD (g/cm^2^)
- Medication (Glucocorticoids, and others)	- Alcohol (3 more units/day)
- Menopause	- Current smoking
- Inflammation	- Glucocorticoids
- Oxidative stress	- Rheumatoid arthritis
- Apoptosis	- Secondary osteoporosis / Diabetes *

^*^In the FRAXPlus application suggesting the entry ‘Diabetes’ instead of Rheumatoid Arthritis or Secondary Osteoporosis
